# Constitutive overexpression of the *TaNF-YB4* gene in transgenic wheat significantly improves grain yield

**DOI:** 10.1093/jxb/erv370

**Published:** 2015-07-27

**Authors:** Dinesh Yadav, Yuri Shavrukov, Natalia Bazanova, Larissa Chirkova, Nikolai Borisjuk, Nataliya Kovalchuk, Ainur Ismagul, Boris Parent, Peter Langridge, Maria Hrmova, Sergiy Lopato

**Affiliations:** University of Adelaide, Australian Centre for Plant Functional Genomics, Urrbrae SA 5064, Australia

**Keywords:** Drought, grain yield, *NF-Y* genes, plant development, protein–protein interactions, wheat

## Abstract

Heterotrimeric nuclear factors Y (NF-Ys) are involved in regulation of various vital functions in all eukaryotic organisms. Although a number of NF-Y subunits have been characterized in model plants, only a few have been functionally evaluated in crops. In this work, a number of genes encoding NF-YB and NF-YC subunits were isolated from drought-tolerant wheat (*Triticum aestivum* L. cv. RAC875), and the impact of the overexpression of *TaNF-YB4* in the Australian wheat cultivar Gladius was investigated. *TaNF-YB4* was isolated as a result of two consecutive yeast two-hybrid (Y2H) screens, where ZmNF-YB2a was used as a starting bait. A new NF-YC subunit, designated TaNF-YC15, was isolated in the first Y2H screen and used as bait in a second screen, which identified two wheat NF-YB subunits, TaNF-YB2 and TaNF-YB4. Three-dimensional modelling of a TaNF-YB2/TaNF-YC15 dimer revealed structural determinants that may underlie interaction selectivity. The *TaNF-YB4* gene was placed under the control of the strong constitutive polyubiquitin promoter from maize and introduced into wheat by biolistic bombardment. The growth and yield components of several independent transgenic lines with up-regulated levels of *TaNF-YB4* were evaluated under well-watered conditions (T_1_–T_3_ generations) and under mild drought (T_2_ generation). Analysis of T_2_ plants was performed in large deep containers in conditions close to field trials. Under optimal watering conditions, transgenic wheat plants produced significantly more spikes but other yield components did not change. This resulted in a 20–30% increased grain yield compared with untransformed control plants. Under water-limited conditions transgenic lines maintained parity in yield performance.

## Highlight

Constitutive overexpression of *NF-YB* in transgenic wheat leads to a 20–30% increase in grain yield compared with wild-type plants (cv. Gladius) without negative changes in seed size or number.

## Introduction

The *Nuclear Factor Gamma* (*NF-Y*) gene family in plants has recently generated much interest due to involvement of family members in plant performance, development, and stress regulation (Petroni *et al.*, 2012; Laloum *et al.*, 2013). NF-Y transcription factors (TFs), also known as histone- or haem-associated proteins (HAPs) or CCAAT-binding factors (CBFs), are heterotrimeric DNA-binding proteins, structurally conserved in all eukaryotes (Mantovani, 1999; Romier *et al.*, 2003). They bind specifically to the CCAAT box (Sinha *et al.*, 1995; Bi *et al.*, 1997), found in ~25% of eukaryotic promoters (Suzuki *et al.*, 2001; Marino-Ramirez *et al.*, 2004). NF-Y TFs are composed of three different subunits named NF-YA (also known as HAP2 or CBF-B), NF-YB (HAP3 or CBF-A), and NF-YC (HAP5 or CBF-C). The NF-Y subunits B and C contain histone-fold domains (HFDs) structurally related to histones H2B and H2A, respectively (Baxevanis *et al.*, 1995). These domains mediate formation of a stable, histone-like heterodimer (Romier *et al.*, 2003). The NF-Y subunit A subsequently binds to the B–C heterodimer to form a heterotrimeric complex, which can specifically recognize the CCAAT box (Sinha *et al.*, 1995).

While most eukaryotic genomes contain only one or two genes encoding each NF-Y subunit (Maity and de Crombrugghe, 1998), NF-Y subunits of vascular plants are generally encoded by gene families with at least 10 members (Riechmann *et al.*, 2000; Cagliari *et al.*, 2014). In hexaploid wheat (*Triticum aestivum* L.), a total of 35 *NF-Y* genes (10 *NF-YA*, 11 *NF-YB*, and 14 *NF-YC*) have been identified (Stephenson *et al.*, 2007), and some of these have been functionally characterized (Stephenson *et al.*, 2010, 2011). Recently the total number of identified *NF-Y* genes in wheat reached 80 (Qu *et al.*, 2015). Since the whole-genome sequence of wheat is not yet completed, the exact number of wheat *NF-Y* genes remains unknown. It was proposed that the opportunity for formation of a large number of heterotrimeric combinations in plants provides substantial plasticity for the involvement of NF-Y complexes in regulation of multiple developmental processes (Edwards *et al.*, 1998).

Subunits of NF-Y TFs were shown to be involved in development and stress responses in different plant species (Edwards *et al.*, 1998; Lotan *et al.*, 1998; Kwong *et al.*, 2003; Lee *et al.*, 2003; Cai *et al.*, 2007; Chen *et al.*, 2007; Nelson *et al.*, 2007; Yazawa and Kamada, 2007; Kumimoto *et al.*, 2008, 2010; Yamamoto *et al.*, 2009; Liu and Howell, 2010; Stephenson *et al.*, 2010, 2011; Wei *et al.*, 2010; Yu *et al.*, 2011; Ni *et al.*, 2013; Qu *et al.*, 2015). A small number of these genes have been evaluated in important cereals such as maize, rice, and wheat (Miyoshi *et al.*, 2003; Nelson *et al.*, 2007; Wei *et al.*, 2010; Ito *et al.*, 2011; Stephenson *et al.*, 2011; Qu *et al.*, 2015). For example, regulation of plant height and yield potential under optimal growth conditions was demonstrated for transgenic rice plants overexpressing the rice gene encoding the HAP3 subunit, *DTH8*, which appears to play an important role as a suppressor in the signal network of photoperiodic flowering (Wei *et al.*, 2010). Constitutive overexpression of the *ZmNF-YB2* gene in transgenic maize significantly improved performance of transgenic plants subjected to mild drought in controlled conditions and during field trials (Nelson *et al.*, 2007). It has recently been demonstrated that the *TaNFYA-B1* gene plays an important role in root development and in nitrogen and phosphorus uptake in wheat (Qu *et al.*, 2015). However, given the large number of *NF-Y* genes in the genome of wheat and the potential for different combinations of these genes to have specific regulatory functions, further characterization of *NF-Y* genes is warranted.

In this work, a wheat homologue of the maize ZmNF-YB2a subunit was cloned using yeast two-hybrid (Y2H) screens of cDNA libraries prepared from tissues of a drought-tolerant wheat cultivar subjected to drought and heat stress. The effects produced by overexpression of one of the isolated wheat genes in the elite Australian wheat cultivar, Gladius, was evaluated.

## Materials and methods

### Plant material

Transgenic plants were generated in a modern Australian wheat variety Gladius. The pedigree of Gladius was as following: RAC-875/Kriachauff//Excalibur/Kukri/3/RAC-875/Krichauff/4/RAC-875//Excalibur/Kukri. The variety was directly developed from a doubled haploid and is, consequently, perfectly homozygous and true breeding. This variety performed well in the hot, dry conditions of southern Australia and is regarded as particularly drought tolerant (Fleury *et al.*, 2010).

### Plasmid construction and plant transformation

A 492bp fragment of the *TaNF-YB4* coding sequence (CDS) was cloned into the pENTR-D-TOPO vector and verified by sequencing using the primers listed in Supplementary Table S1 available at *JXB* online, and subcloned into the vector pUbi (Eini *et al.*, 2013) by recombination. The resulting construct was designated as pUbi-TaNF-YB4. The pUbi-TaNF-YB4 construct was linearized using the unique *Pme*I restriction site and co-transformed with a selection marker cassette (pUbi-Hyg-*nos*) into the Australian elite wheat cv. Gladius, using a biolistic bombardment method described by Kovalchuk *et al.* (2009) and Ismagul *et al.* (2014). Transgene integration was confirmed by PCR using a forward primer derived from the 3′ end of the transgene CDS and a reverse primer from the 5′ end of the *nos* terminator (Supplementary Table S1), for 17 independent transgenic events. Transgene copy number was estimated by quantitative PCR as described by Fletcher (2014).

### Yeast two-hybrid screen

The CDS of *ZmNF-YB2a* (accession no. NP_001105435) was cloned into the vector pGBKT7 (Invitrogen, Victoria, Australia) for Y2H screening. *ZmNF-YB2a* was used because maize *ZmNF-YB2* cDNA could not be isolated based on the sequence submitted to public databases by Nelson *et al.* (2007). The only protein sequence difference between the two is an insert of seven amino acid residues (GKTIPAN) in the HFD of ZmNF-YB2, which is absent in ZmNF-YB2a and similar NF-YB subunits from wheat and other grasses. ZmNF-YB2a was used as bait to screen WGL and WENDL (Lopato *et al.*, 2006), WHSL (Eini *et al.*, 2013), WGD (developing grain at 0–6 days after pollination collected from the wheat cv. RAC875 subjected to drought at flowering), and WRDL (roots of wheat cv. RAC875 seedlings grown in soil and subjected to drought) cDNA libraries as previously described (Eini *et al.*, 2013). A large number of independent clones containing full-length or partial coding regions of the *TaNF-YC15* cDNA were isolated. Because of self-activating properties of the full-length TaNF-YC15, a truncated version of the protein beginning at residue Ala97 was used to re-screen the same cDNA libraries. Clones containing inserts encoding two different full-length NF-YB TFs, designated as TaNF-YB2 (18 independent clones) and TaNF-YB4 (22 independent clones), were isolated.

### Phylogenetic analysis of NF-Y subunits containing histone fold domains

The amino acid sequences of 53 NF-YB and 32 NF-YC proteins were aligned with Clustal Omega (Sievers *et al.*, 2011) and alignments were further inspected by PROMALS3D (Pei *et al.*, 2008) and the Alignment Annotator (Gille *et al.*, 2014). Unrooted phylogenetic trees, based on crude distance measures, were visualized in TreeView (Page, 1996). GenBank accession numbers of protein sequences included in the phylogenetic trees can be found in the Supplementary data.

### Construction of a three-dimensional (3D) model of the TaNF-YB2/TaNF-YC15 dimer

A 3D model was constructed by using mapping monomeric threading alignments to protein–protein interactions based on oligomeric entries in the Protein Data Bank (PDB) (Guerler *et al.*, 2013). The crystal structure of the NF-YB/NF-YC dimer from *Homo sapiens* (PDB accession 1N1J, chains A/B, designated as 1N1J:A/1N1J:B) (Romier *et al.*, 2003) was identified as a suitable quaternary assembly template for structural modelling. The full-length TaNF-YB2 and TaNF-YC15 sequences were analysed by SMART (Letunic *et al.*, 2012), ProDom (Bru *et al.*, 2005), and SBASE (Vlahovicek *et al.*, 2002) to determine domain arrangements and the positions of HFDs. The TaNF-YB2 and TaNF-YC15 sequences were aligned with those of 1N1J:A and 1N1J:B, respectively, using LOMETS (Wu and Zhang, 2007), and the alignment quality was checked by the Alignment Annotator (Gille *et al.*, 2014) and PSIPRED (Buchan *et al.*, 2013) to confirm that secondary structures remained undisturbed. The aligned sequences were submitted to SPRING (Guerler *et al.*, 2013) and the most suitable model of TaNF-YB2/TaNF-YC15, evaluated by structural criteria, was selected from a library of six models. The model was minimized using AMBER99 force field to achieve optimal stereochemical parameters. A Ramachandran plot of the optimized model indicated that 100% of the residues were in the most favoured, additionally allowed, and generously allowed regions, when excluding glycine and proline residues. The overall G-factor values evaluated by PROCHECK (Laskowski *et al.*, 1993) were 0.55 and 0.11 for 1N1J:A/1N1J:B and TaNF-YB2/TaNF-YC15, respectively. The *Z*-score values, deduced from Prosa2003 (Sippl, 1993) and reflecting combined statistical potential energy, were –5.21 and –5.12 for 1N1J:A/1N1J:B and TaNF-YB2/TaNF-YC15, respectively. The root-mean-square deviation (RMSD) values between 1N1J:A/1N1J:B (165 residues) and TaNF-YB2/TaNF-YC15 (169 residues) determined with a PyMol ‘super’ algorithm were 1.1 Å for 164 residues in Cα positions. Images of structural models were generated in the PyMol Molecular Graphics System, Version 1.3 Schrödinger, LLC.

### Genotyping and transgene expression analyses

Plant DNA was extracted from leaf tissue using a freeze-drying method described by Shavrukov *et al.* (2010). Individuals were genotyped for the presence of the transgene. Transgene copy number was estimated by efficiency-adjusted real-time quantitative PCR (qRT-PCR), using *nos* terminator-specific primers (Supplementary Table S1 at *JXB* online; Kovalchuk *et al.*, 2013). For template loading normalization, primers and a probe complementary to a portion of the single-copy endogenous *Puroindoline-b* (*Pin-b*) gene were used (Li *et al.*, 2004). The oligonucleotide sequences were: forward 5′-ATTTTCCAGTCACCTGGCCC-3′; reverse 5′-TGCTATCTGGCTCAGCTGC-3′; and dual-labelled TaqMan probe 5′-CAL fluor Gold 540-ATGGTGGAAGGG CGGCTGTGA-BHQ1-3′.

Total RNA was isolated from leaf tissue using the Direct-zol RNA MiniPrep (Zymo Research Corporation, ACT, Australia). Transgene expression was confirmed using reverse transcription–PCR (RT–PCR) and transgene-specific primers (Supplementary Table S1 at *JXB* online). For northern analysis of the transgene expression, RNA was electrophoretically separated in a 1.3% agarose gel containing 6% formaldehyde, transferred to a nylon membrane, and hybridized with ^32^P-labelled DNA probes according to the protocol described by Church and Gilbert (1984).

### Gene expression analysis of native *NF-Y* genes in wheat

qRT-PCR analyses of gene expression in various tissues of non-transgenic wheat were performed on cDNA samples as described by Burton *et al.* (2008). Four control genes were used to normalize the qRT-PCR data (primers in Supplementary Table S1 at *JXB* online) as described by Fletcher (2014). Samples included cDNA developmental tissue series and were prepared from different tissues of *T. aestivum* cv. Chinese Spring (Morran *et al.*, 2011). A dehydration cDNA series was prepared from detached leaves of 4-week-old seedlings of drought-tolerant wheat cv. RAC875 (Kovalchuk *et al.*, 2013), which were incubated for 0, 0.5, 1, 2, 4, 5, and 7h at room temperature (23 °C) in opened 15ml plastic tubes, before snap-freezing in liquid nitrogen for RNA extraction. There were three biological replicates in each qPCR experiment, which was repeated four times.

### Analysis of transgenic plants

#### Experiment 1

Three lines estimated to have a single copy of the transgene (L3, L4, and L5) and one line with two copies (L6) were selected for preliminary characterization of plant phenotypes and seed multiplication. Six T_1_ plants for each line were grown in 15cm diameter pots, one plant per pot, in coco-peat soil in a greenhouse (24/16 °C, day/night temperature; 16h day) under well-watered conditions. Untransformed wild-type (WT) plants were also grown for the comparison. Plant height, number of tillers and spikes, plant biomass, flowering time, seed number, and seed weight were recorded for each plant. Transgene copy number and transgene expression levels (Supplementary Fig. S1 at *JXB* online) were also determined, and null segregants were excluded from the analyses.

#### Experiment 2

Six T_2_ sub-lines (L3-5, L4-2, L4-4, L5-4, L6-2, and L6-3) derived from four independent T_1_ lines were selected for a second experiment under a controlled water regime, conducted in two large containers (190×68×60cm) filled with a 1:1:1 mix of coco-peat, river sand, and clay soil collected near Adelaide (South Australia). The soil was re-used for two seasons, and 1/5 of coco-peat soil was added before a new sowing to compensate for any loss of nutrients. In general, nutrient supply in this soil was relatively low but comparable with non-fertilized field soil. Plants were grown in rows, with eight plants per row for each line and the WT with three randomized blocks in each container, comprising in total 18–26 biological replicates for each line as well as for the WT and null segregant lines. Experimental plants were flanked by a border row of WT plants on each short side of the container. The experimental design was identical for each container. No significant differences were found in plant growth between the three blocks in preliminary experiments (data not shown), and therefore all replicates for each line, WT, and nulls were used to calculate means and standard errors for the measurements.

Containers were watered every second day. The watering was withdrawn in the droughted container when the majority of plants reached tillering; watering was continued in the well-watered container until the end of grain maturation. A soil water potential of –0.3±0.05MPa was reached in the droughted container a short time before flowering, and plants were kept under slowly increasing drought until ~10 d after the end of flowering. Soil water content was monitored by the system, Magpie-3 (Measuring Engineering Australia), where data for water content in the soil were regularly collected by sensors from each container and from three levels of depth in the soil: 15, 30, and 45cm. Curve graphs of droughted and well-watered bins were automatically recorded. When water potential in the droughted container reached –0.5MPa, watering re-commenced and soil moisture was restored to a level similar to the well-watered container (Supplementary Fig. S2 at *JXB* online).

Measurements of yield-related plant growth traits and yield components were taken at harvest, as for Experiment 1. Leaf material sampled from all plants was used for genotyping and analysis of gene expression. Five of the six T_2_ sub-lines (L3-5, L4-2, L4-4, L5-4, and L6-3) were identified as homozygous for the transgene (Supplementary Fig. S1 at *JXB* online). In the segregating heterozygous sub-line (L6-2), null segregants were excluded from the analyses, as were progeny of line L3 due to a large number of dead plants (Table 1).

**Table 1.** *T*_*1*_ *progeny of* TaNF-YB4 *transgenic lines analysed in large containers*

Growth conditions	Lines	WT	Null	L3-5^a^	L4-2^*b*^	L4-4^*b*^	L5-4^*b*^	L6-2	L6-3^*b*^
Trangene copy number		0	0	1	1	1	1	2	1
Well-watered	Total no. of plants	21	22	10	21	28	28	28	28
Well-watered	No. of dead plants	3	2	10	0	4	3	1	1
Well-watered	No. of nulls	–	22	0	0	0	0	4	0
Well-watered	No. of plants with expressed transgene^*c*^	–	0	10	21	24	25	23	27
Drought	Total no. of plants	24	23	9	24	24	24	24	24
Drought	No. of dead plants	2	1	9	2	1	2	0	2
Drought	No. of nulls	–	23	0	0	0	0	4	0
Drought	No. of plants with expressed transgene	–	0	9	22	23	22	20	28

^*a*^ Progeny of sub-line 3-5 demonstrated a poor growth and high mortality, and therefore it was not shown in Fig. 5 or used in further experiments

^*b*^ Homozygous sub-lines.

^*c*^ Only data for plants with the expressed transgene were used for generation of the graphs in Fig. 6.

#### Experiment 3

T_3_ wheat sub-lines were grown in 22cm diameter pots, four plants per pot, including one WT and three transgenic plants. Transgenic plants included representatives of two confirmed and one possible homozygous sub-line (L4-4-51, L5-4-52; and L6-3-31). A coco-peat soil amended with five levels (0, 0.7, 1.4, 2.1, and 2.8gram per litre of soil) of slow-release complete fertilizer (Osmocote Exact Mini, Everris International, Australia) was used in this experiment. The nutrient content of the soil was in the range of medium to high, and comparable with the levels reached in well-fertilized field soil. Three pots of plants (replicates) were grown for each level of fertilizer, except for the unamended treatment, for which there were six pots. Analysis of yield components was performed as described above for Experiments 1 and 2.

### Statistical treatment of data

Single-factor analyses of variance (ANOVAs) and Student *t*-tests (unpaired, two-tails) from Microsoft Excel software were applied for statistical analyses of data from the plant phenotyping experiments. Numbers of biological replicates were 18–26 (Experiment 2) and 3–6 (Experiment 3).

## Results

### Isolation of wheat genes encoding NF-YB subunits

In order to identify and isolate cDNA clones encoding wheat NF-YB proteins homologous to maize ZmNF-YB2, a two-step Y2H screen was performed. Full-length ZmNF-YB2a protein (accession no. NP_001105435) was used to screen cDNA libraries prepared from different wheat tissues of drought-tolerant wheat cv. RAC875 subjected to drought and heat stresses.

**Fig. 1.**
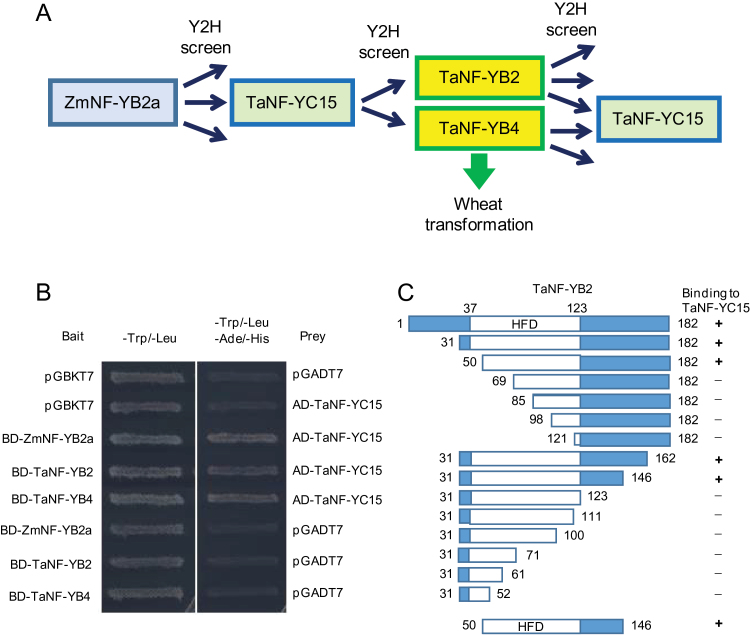
Cloning strategy, confirmation of interactions, and identification of protein segments responsible for the interaction of wheat NF-YB and NF-YC subunits. (A) Schematic representation of the yeast two-hybrid screens and cloned products. Open arrows indicate cloned genes that are not discussed herein. (B) Confirmation of interaction of TaNF-YC15 with maize and wheat NF-YB proteins in a reciprocal direction using re-cloned coding regions (5′- and 3′-untranslated regions removed) of cloned wheat cDNAs. (C) Identification of the smallest possible TaNF-YB2 segment interacting with TaNF-YB15 by using a set of N-terminal and C-terminal truncations. (This figure is available in colour at *JXB* online.)

The screen resulted in isolation of several TFs and transcription-related proteins (data not shown). The most abundant of the isolated sequences was from an unreported wheat gene encoding the NF-Y subunit C, designated as TaNF-YC15. To prevent self-activation during the Y2H screen, TaNF-YC15 was truncated at the N-terminal end. Truncation did not affect the HFD, important for interactions with NF-YB proteins. Y2H screens with TaNF-YC15 resulted in isolation of large numbers of independent clones encoding two different NF-YB subunits identical to TaNF-YB2 and TaNF-YB4, which were previously described by Stephenson *et al.* (2007) (Fig. 1A). Full-length coding regions of isolated wheat *NF-YB* cDNAs were re-cloned into the bait vector, and interaction of TaNF-YB2 and TaNF-YB4 with TaNF-YC15 was demonstrated in a reciprocal way (Fig. 1B). In addition, it was found that TaNF-YC15 is the most abundant among several NF-YC subunits isolated in Y2H screens using TaNF-YB2 and TaNF-YB4 as bait proteins (data not shown).

**Fig. 2.**
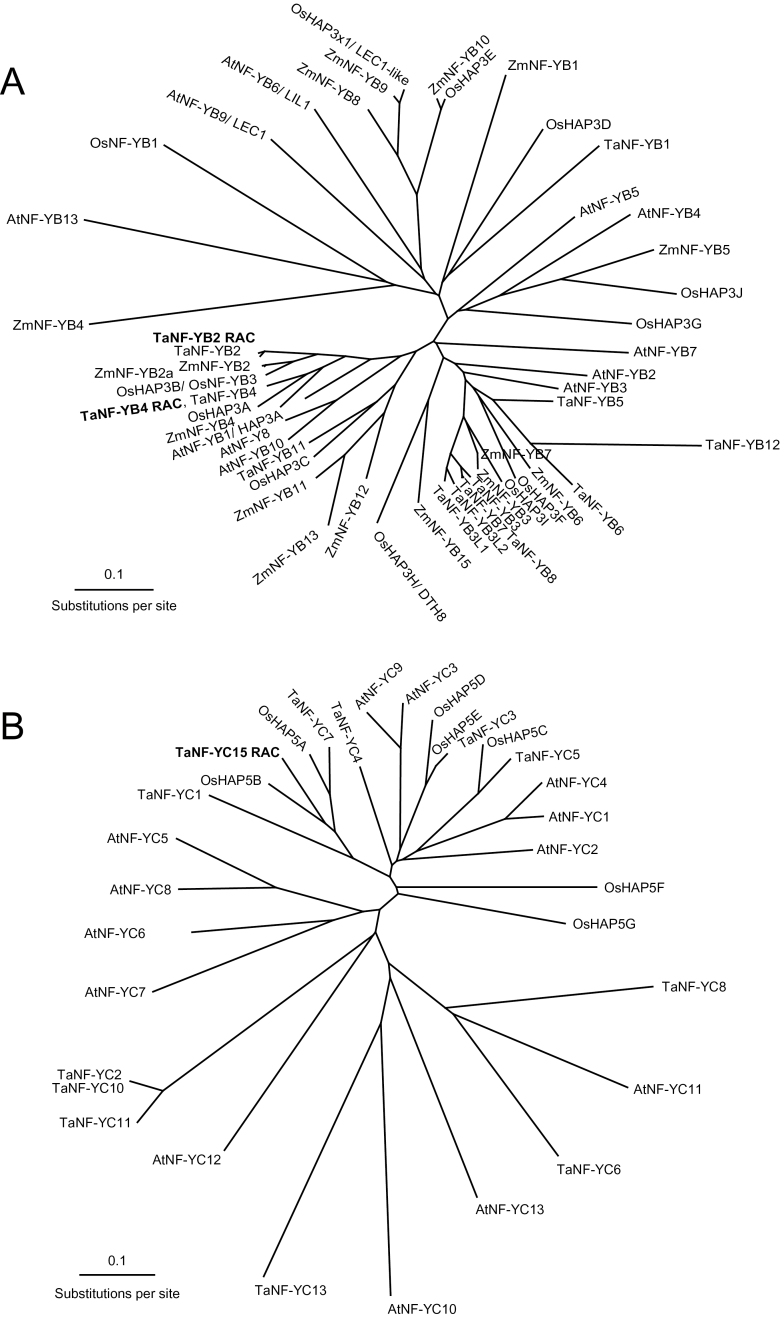
An unrooted radial phylogenetic tree of (A) NF-YB, and (B) NF-YC TFs from wheat, rice, maize, and *Arabidopsis*. Sequences of 52 NF-YB and 32 NF-YC proteins were aligned using ProMals3D (Pei *et al.*, 2008) with branch lengths drawn to scale. Two-letter prefixes at sequence identifiers indicate species of origin. GenBank accession numbers of all proteins are listed in the Supplementary data at *JXB* online. Maize sequences that have no names in GenBank annotations were given the same number as wheat proteins grouped to the same clades (where it was possible). New wheat proteins with a high level of identity to previously published proteins were designated with the same name and an additional letter and number, e.g. L at the end of the name. TFs isolated in this work (in bold) are marked with three additional letters (RAC is shorthand for cv. RAC875).

The phylogenetic relationships between NF-YB and NF-YC protein sequences from wheat, maize, rice, and *Arabidopsis* were revealed in unrooted trees constructed using Clustal Omega (Blackshields *et al.*, 2010) (Fig. 2). The tree for the NF-YB sequences shows that both TaNF-YB2 and TaNF-YB4 belong to two different branches of the same protein clade, and that TaNF-YB2 and ZmNF-YB2 are the products of orthologous genes as they are grouped together. The TaNF-YB4 protein is grouped with a protein from maize, which is designated as ZmNF-YB4, while two rice proteins are grouped within these two branches. In contrast, the *Arabidopsis* AtNF-YB1 protein has a high level of identity to proteins from both branches containing TaNF-YB2-like and TaNF-YB4-like proteins, and two other proteins, AtNF-YB8 and AtNF-YB10, have much lower levels of identity to TaNF-YB2 (59% and 63%, respectively) or TaNF-YB4 (58% and 61%, respectively), but still are grouped into the same clade (Fig. 2A). The isolated TaNF-YB2 protein has a single amino acid residue difference from that submitted to the GenBank database (accession no. BT009078). This may reflect the fact that these two proteins originate from different wheat cultivars. No differences were found in the protein sequences for TaNF-YB4 isolated from cv. RAC875 in this study and the GenBank entry for TaNF-YB4 (accession no. BT009393).

TaNF-YC15 has a high level of protein sequence identity to TaNF-YC7 (accession no. AED95949). Both proteins belong to the same clade together with orthologous proteins from rice and the more distant TaNF-YC1 (accession no. CD888515). No proteins from *Arabidopsis* were grouped in the same clade (Fig. 2B).

### TaNF-YB2 and TaNF-YB4 interact with TaNF-YC15

To explain why only two out of more than a dozen wheat NF-YB factors interacted with TaNF-YC15, the essential segment of the TaNF-YB2 protein was mapped in a Y2H assay using a series of truncated forms of TaNF-YB2 (Fig. 1C). The identified minimal protein segment, which interacted with the NF-YC subunit, contained a significant proportion of the HFD, as well as additional amino acid residues adjacent to the C-terminal part of the HFD.

**Fig. 3.**
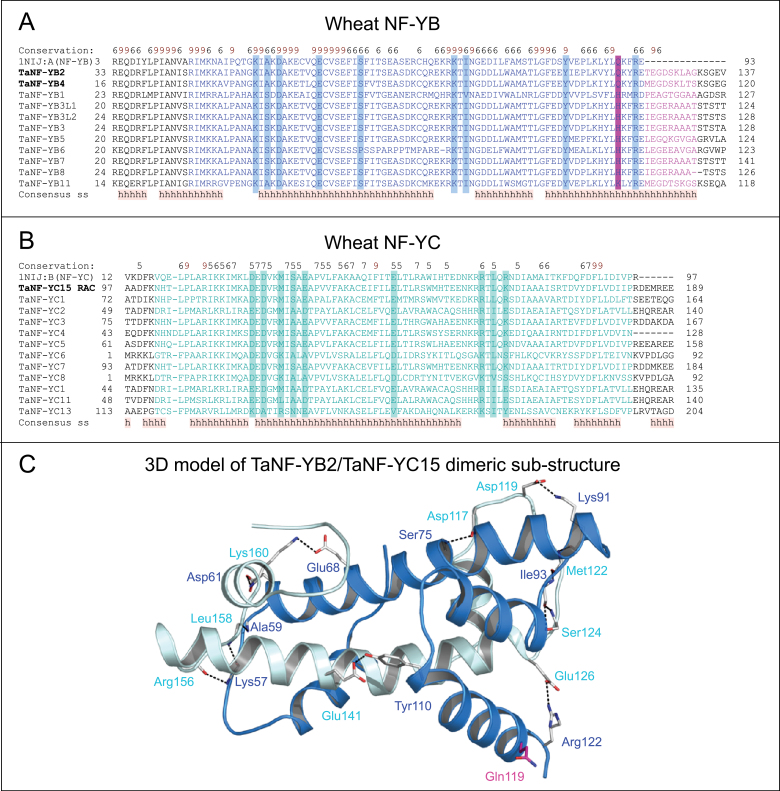
Molecular model of the wheat TaNF-YB2/TaNF-YC15 dimeric substructure and structural bioinformatics of wheat NF-YB and NF-YC transcription factors. (A) A multiple sequence alignment of HFDs (coloured in blue) of 11 TaNF-YB from wheat. The sequence of a human NF-YB (PDB accession 1NIJ:A also designated NF-YB) is also included. Protein sequences were aligned with ProMals3D (Pei *et al.*, 2008). Predicted consensus α-helices (h) are marked. Conservation of residues on a scale of 9–6 is shown at the top of the diagram. Predicted residues involved in binding to HFD of NF-YC are marked in blue boxes. An additional nine residues at the C-terminus of TaNF-YB are in magenta, and these residues might also participate in binding of TaNF-YC proteins. Conserved glutamine or histidine residues at the C-terminal regions are shaded in magenta. TaNF-YB2 and TaNF-YB4 isolated in this work are in bold. (B) A multiple sequence alignment of HFDs (cyan) of 12 TaNF-YCs from wheat. The sequence of a human NF-YC (PDB accession 1NIJ:B; also designated NF-YC) is also included. Predicted residues involved in binding to the HFD of NF-YB are marked in cyan boxes. TaNF-YC15 isolated in this work is in bold. Protein sequences were aligned as described in (A). Other annotations are as specified in (A). (C) Molecular model of the wheat TaNF-YB2/TaNF-YC15 dimeric substructure (sequences highlighted in bold in A and B). Secondary structures are shown in cartoon representations (blue for TaNF-YB, cyan for TaNF-YC). Residues participating in binding (TaNF-YB to TaNF-YC: Lys57, Ala59, Asp61, Glu68, Ser75, Lys91, Ile93, Tyr110, and Arg122; TaNF-YC to TaNF-YB: Asp117, Asp119, Met122, Ser124, Glu126, Gly141, Arg156, Leu158, and Lys160) involved in dimer formation are shown in sticks and atomic colours. Gln119 in close proximity to Arg122 is in magenta. Distances between interacting residues are shown in black dashed lines and vary between 2.7 Å and 3.5 Å.

The HFD portions of 11 NF-YB and 12 NF-YC protein sequences from wheat were aligned with those of human NF-YB and NF-YC, showing an exceptionally high level of conservation (Fig. 3A, B). Furthermore, a comparison of the modelled TaNF-YB2/TaNF-YC15 wheat structural dimer with the crystal structure of the human NF-YB/NF-YC (1N1J:A/1N1J:B) subcomplex indicated that the two substructures were highly similar. Both complexes that interacted through HFDs showed features that were common to this class of proteins. There were high levels of sequence identity/similarity (76.9/98.9% and 76.9/93.6% for A/B and B/C chains, respectively) (Smith and Waterman, 1981) and structural correspondence (RMSD values 1.1 Å) between wheat and human NF-YB/NF-YC complexes, indicating that this structural fold has not undergone significant diversification during evolution. The residues involved in dimerization of TaNF-YB2/TaNF-YC15 included nine residues each in chain B (Lys57, Ala59, Asp61, Glu68, Ser75, Lys91, Ile93, Tyr110, and Arg122) and chain C (Asp117, Asp119, Met122, Ser124, Glu126, Gly141, Arg156, Leu158, and Lys160) (Fig. 3C). All nine residues in TaNF-YB2 and eight out of the nine residues in TaNF-YC15 were conserved compared with human NF-YB/NF-YC, except for Lys160 that was substituted with Arg75 in human NF-YC (Fig. 3B, C).

All residues of TaNF-YB2 HFD (Fig. 3) were present in the minimal segment of TaNF-YB2 (residues 50–146; Fig 1C) that were required for interaction with TaNF-YC15 in Y2H assay. A truncated variant of TaNF-YB2 with a C-terminal Glu123 (underlined in the motif QKYR*E*; Fig. 3B) lost the ability to bind TaNF-YC15. This suggested a minimal length for TaNF-YB2 protein to ensure correct folding of TaNF-YB2 (Fig. 3B), and allowing Arg122 (that precedes Glu123) to recover its proper protonation state and function during formation of the TaNF-YB2/TaNF-YC15 complex. Gln119 of wheat TaNF-YB2 was located in close proximity to Arg122 in TaNF-YB2 and was also present in TaNF-YB4 (and human NF-YB; Fig. 3B), but was substituted in all other TaNF-YBs by a histidine residue.

**Fig. 4.**
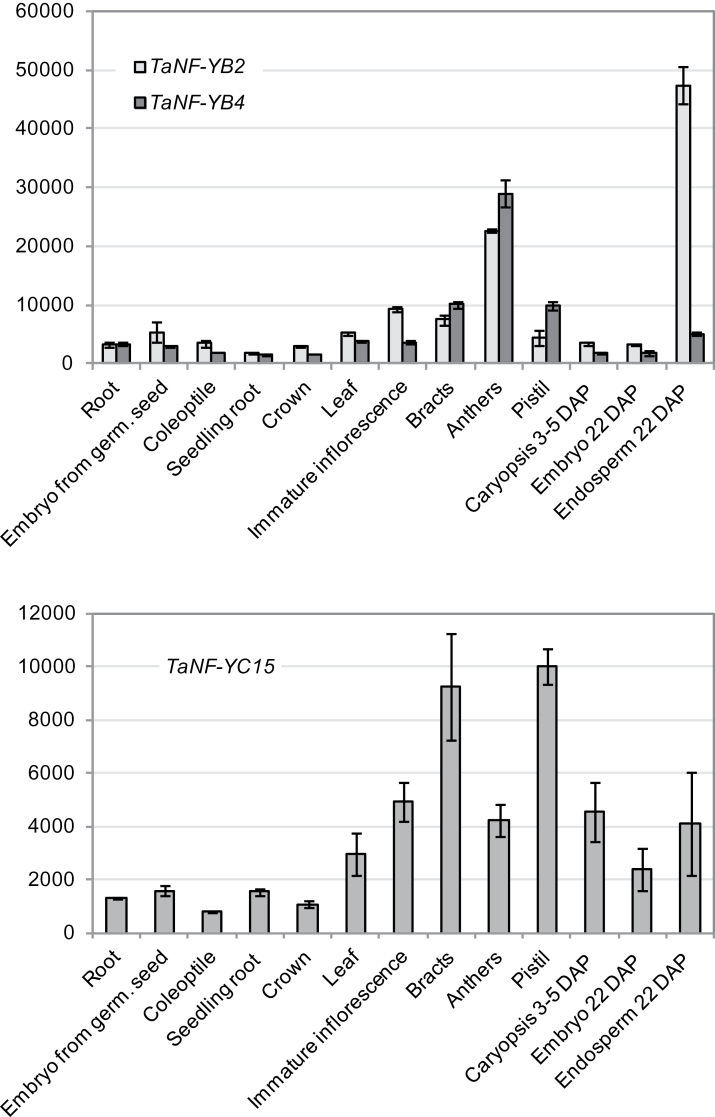
Expression of the *TaNF-YB2*, *TaNF-YB4*, and *TaNF-YC15* genes in different wheat tissues (cv. RAC875) in the absence of stress. Levels of expression were detected by qRT-PCR and are shown as normalized transcription levels in arbitrary units.

Further indirect evidence that wheat TaNF-YC15 acts in concert with either TaNF-YB2 or TaNF-YB4 came from gene expression analyses of plant tissues treated under a variety of conditions. Expression levels of *TaNF-YC15* in these wheat tissues correlated well with levels of *NF-YB* transcripts, particularly with *TaNF-YB4* (Fig. 4).

### Expression levels of isolated NF-Y transcription factors in different tissues, during slow developing drought and rapid dehydration

Transcript levels of *TaNF-YB2*, *TaNF-YB4*, and *TaNF-YC15* genes were analysed by qRT-PCR in different tissues of unstressed wheat plants and in leaves from plants subjected to abscisic acid (ABA) treatment, drought, or rapid dehydration. Expression of both *NF-YB* factors was detected in all tested tissues of unstressed wheat plants. Higher levels of expression were observed in reproductive tissues and particularly in anthers, where levels of expression of both genes were 5- to 6-fold higher than in other tissues (Fig. 4A). Correlation between *TaNF-YB2* and *TaNF-YB4* levels of expression was observed across most tissue types. The most significant difference between expression levels of *NF-YB* genes was found in mature endosperm, where the number of *TaNF-YB2* transcripts was ~9-fold higher than the number of *TaNF-YB4* transcripts (Fig. 4A). Expression levels of *TaNF-YC15* in different wheat tissues correlated well with levels of *NF-YB* transcripts, particularly with *TaNF-YB4*, with one exception (Fig. 4B). In contrast to *NF-YB* genes, level of *TaNF-YC15* expression in anthers was lower than in bracts and pistil. Overall, the *TaNF-YC15* gene was less highly expressed than *TaNF-YB2*/*YB4*.

**Fig. 5.**
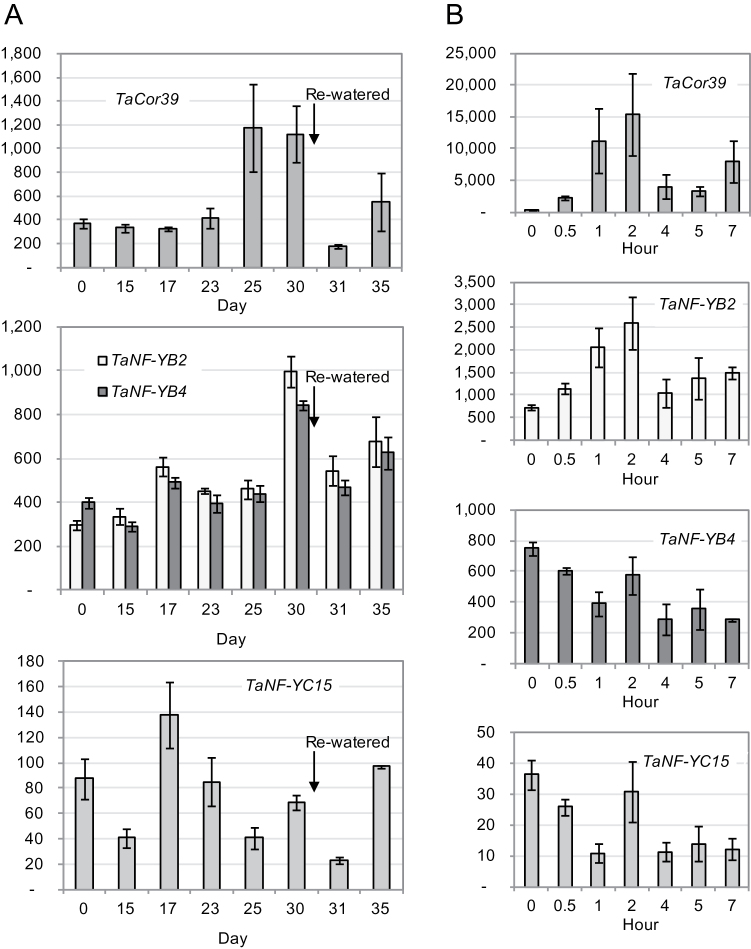
Expression of *NF-Y* genes under drought and rapid dehydration. (A) Drought-inducible expression of the *TaNF-YB2*, *TaNF-YB4*, and *TaNF-YC15* genes in leaves of 4-week-old seedlings. (B) *NF-Y* expression in detached leaves during dehydration at room temperature. The stress-inducible *TaCor39* gene was used as a positive control. Levels of expression were detected by qRT-PCR and are shown as normalized transcription levels in arbitrary units.

Neither *TaNF-YC15* nor *TaNF-YB* was induced by ABA treatment (data not shown). In a drought cDNA tissue series, the three *NF-Y* genes showed two periods of induction. The first induction point occurred on the 17th day of drought treatment, when plants had just started to sense drought, and the second induction point happened at the wilting point, 30 d after watering was withheld (Fig. 5A). A control gene known to be drought stress inducible, *TaCor39* (Kovalchuk *et al.*, 2013), showed a single increase in expression across the same tissue series, at 30 d after cessation of watering (Fig. 5A). Following moderate re-watering, expression levels of all tested genes decreased, but began to increase again after a further 4 d as the soil profile dried out (day 35).

In contrast to slowly developing drought, the rapid dehydration of detached leaves at room temperature led to different patterns of *TaNF-YB2* and *TaNF-YB4* expression. While *TaNF-YB2* was induced by 3-fold, *TaNF-YB4* levels decreased with dehydration. Interestingly, the patterns of expression of *TaNF-YB4* and *TaNF-YC15* in dehydrating leaves were nearly identical (Fig. 5B). The *TaCor39* gene was used as a dehydration-inducible control gene to confirm the quality of the dehydration cDNA series.

### Evaluation of transgenic wheat plants overexpressing *TaNF-YB4* in well-watered, droughted, and nutrient-depleted conditions

Transgenic wheat plants (cv. Gladius) were generated with constitutive overexpression of *TaNF-YB4* driven by the maize ubiquitin promoter. In the preliminary evaluation of plant performance (T_1_ generation), three from four transgenic lines with one or two copies of the transgene produced more grain compared with control plants, without substantial changes in other yield components (data not shown).

In the second experiment, two large containers were used for phenotyping of a large number of transgenic plants (18–28 T_2_ individuals from each of six T_2_ sub-lines, L3-5, L4-2, L4-4, L5-4, L6-2, and L6-3; see Table 1). All of these except L6-2 were determined to be homozygous for the transgene. Progeny of the sub-line L3-5 grew more slowly than other plants and showed a high level of mortality (Table 1), possibly due to a very high level of transgene expression (>10-fold higher than in other lines; data not shown), or as a result of effects due to the position of transgene integration. This subline was not considered for further analysis.

**Fig. 6.**
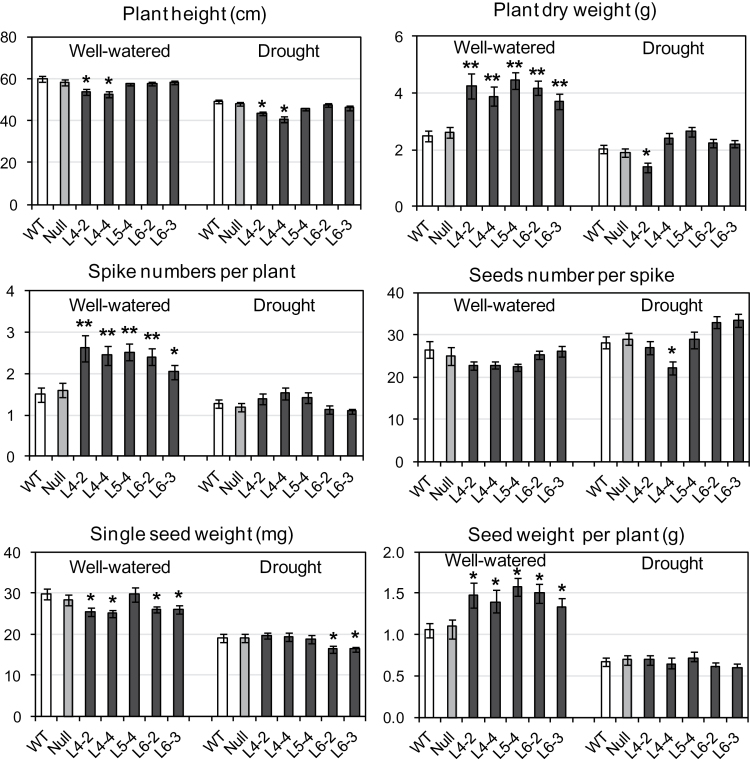
A comparison of phenotypes and grain yields of transgenic and control (WT and mull segregant) wheat plants grown in large containers under well-watered conditions and constantly increasing drought (see Supplementary Fig. S2A at *JXB* online). From 16 to 28 WT, nulls, and transgenic T_2_ plants with confirmed transgene expression for each sub-line of three independent lines were used in the experiment (see Table 1). All sub-lines except line 6-2 were confirmed to be homozygous. Differences between transgenic lines and both WT and null plants were tested in the unpaired Student’s *t*-test (*P<0.05; **P<0.01).

Under well-watered conditions, the transgenic T_2_ plants yielded up to 30% more mass of grain per plant (Fig. 6). Supporting the initial observations, all five analysed transgenic lines showed significant increases in the number of spikes, vegetative biomass, and grain weight per plant compared with control plants (Fig. 6). There was a slight reduction in single-grain weight for four of the lines. Thus, the increased grain yield was attributable to an increased number of spikes per plant (Fig. 6). No yield advantage was observed for transgenics compared with control plants under mild drought.

**Fig. 7.**
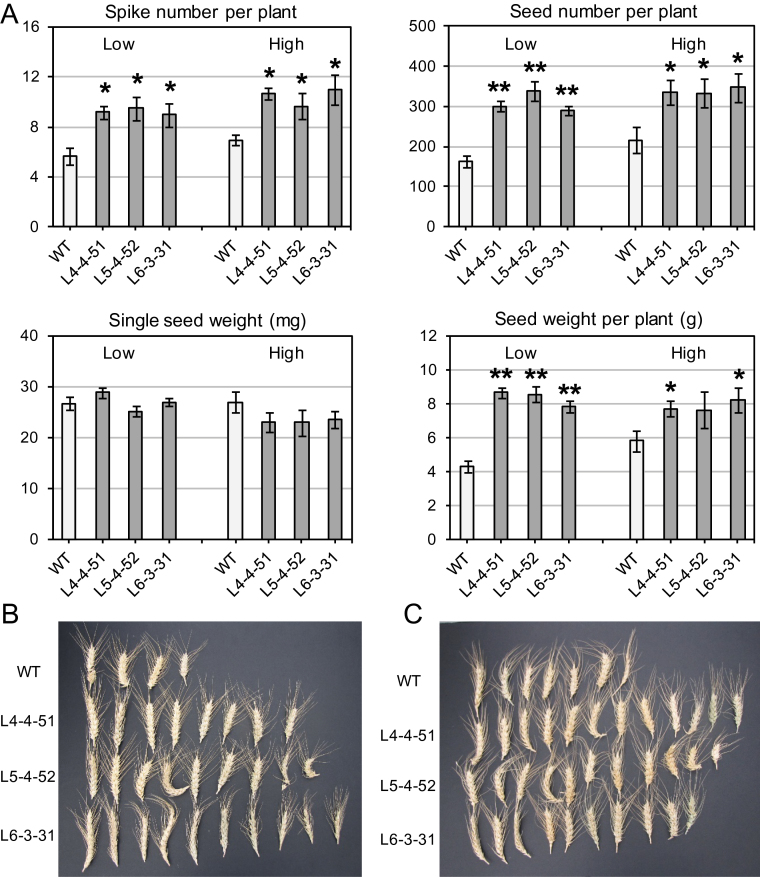
A comparison of phenotypes and grain yields of transgenic and control (WT) wheat plants grown in soil mixes (A), either without added fertilizer (marked as ‘low’), or with the addition of a complete, slow-release fertilizer (marked as ‘high’) (see Table 2). One WT plant and one T_3_ transgenic plant for each of three homozygous sub-lines with confirmed transgene expression were grown in the same pot. Six pots were used for the soil mix without added fertilizer, and three pots for the fertilized treatments. Differences between transgenic lines and WT plants were tested in the unpaired Student’s *t*-test (*P<0.05; **P<0.01). Images of spikes from plants grown in separate pots: (B) without addition and (C) with addition of a complete slow-release fertilizer. (This figure is available in colour at *JXB* online.)

Preliminary observations of plant performance in nutrient-depleted media indicated that NF-Y factors may impart the greatest yield advantage in poor soils. Thus, a third growth experiment was conducted using *TaNF-YB4* homozygous transgenic lines (T_3_) to investigate this hypothesis. Plants were grown in a coco-peat soil either with no addition or with four different rates of Osmocote, a slow-release complete fertilizer. Increases in grain yield of the transgenic plants compared with control plants were evident across all fertilizer treatments (Fig. 7; Table 2). However, there was also an interaction between fertilizer rate and the yield advantage of transgenic lines. Control plants showed a 25% increase in yield in response to fertilizer addition. In contrast, no positive effect of additional fertilizer on the grain yield of transgenic lines was observed (Fig. 7).

**Table 2.** *Influence of different amounts of the slow-release fertilizer Osmocote Exact Mini on yield components of WT (wheat cv. Gladius) and three homozygou*s TaNF-YB4 *transgenic lines*

Line	Additional Osmocote (gram per litre of soil)				
	0	0.7	1.4	2.1	2.8
Spike number per plant					
WT*	5.7±0.7	6.1±0.8	6.3±0.7	6.5±0.5	6.9±0.4
L4-4-51	9.2±0.5	10.7±0.4	10.3±1.2	11.0±0.7	10.7±0.5
L5-4-52	9.5±0.9	9.7±1.5	9.7±0.4	10.3±1.1	9.7±1.0
L6-3-31	9.0±1.0	10.0±1.4	11.0±0.9	11.0±0.7	11.0±1.2
Seed number per plant					
WT*	162.3±14.6	169.0±31.3	189.7±20.2	206.3±26.0	215.7±32.2
L4-4-51	301.3±12.3	291.3±11.7	340.7±28.0	341.0±13.7	336.3±30.1
L5-4-52	339.8±24.0	286.7±32.3	269.3±30.6	298.0±33.4	333.3±36.6
L6-3-31	290.7±12.3	287.7±21.8	332.7±29.5	324.3±28.8	348.0±35.8
Seed weight per plant (g)					
WT*	4.33±0.34	4.29±0.64	4.70±0.49	5.53±0.65	5.83±0.64
L4-4-51	8.69±0.31	8.28±0.62	8.61±0.70	8.28±0.21	7.73±0.45
L5-4-52	8.57±0.48	8.02±0.81	7.88±0.87	7.76±0.35	7.65±1.10
L6-3-31	7.85±0.38	7.82±0.88	8.29±0.62	8.29±0.88	8.22±0.75

Data are presented as means (*n*=3) ±SE with the exception for the first treatment (denoted as 0), where *n*=6.

WT plants (*) were statistically different from each of three lines at least for *P*<0.05 for each treatment and each trait.

## Discussion

### ZmNF-YB2, TaNF-YB2, and TaNF-YB4 bind to TaNF-YC15

Plant genomes contain >10 genes for each of three subunits of NF-Y proteins (Riechmann *et al.*, 2000), making it difficult to identify the specific subunits that may come together to form heterotrimeric complexes. However, Y2H screening methods such as employed in this study allow the direct identification of interacting proteins from libraries of expressed genes. ZmNF-YB2a was selected to be used as starting bait in sequential Y2H screens, to find related wheat NF-Y subunits and other proteins. ZmNF-YB2 has been demonstrated to increase grain yield when constitutively overexpressed in maize, particularly under drought (Nelson *et al.*, 2007). Aiming to identify related genes possibly implicated in drought stress tolerance in wheat, Y2H cDNA libraries prepared from different tissues of the Australian drought-tolerant wheat cultivar RAC875 were screened, including tissues from plants subjected to drought or to combined drought and heat stresses.

In the first round of the Y2H screening, a novel wheat protein, TaNF-YC15, encoded by DNA inserts of most clones isolated with ZmNF-YB2a as bait, was identified. Then a truncated N-terminal version of TaNF-YC15, containing the full-length HFD that is important for protein–protein interactions (Fig. 3B, C), was used to screen for interacting proteins from the same wheat tissue libraries. In these Y2H screens, only two types of NF-YB subunits were found, TaNF-YB2 and TaNF-YB4, full-length cDNAs of which were identified in the majority of isolated independent clones. Both proteins had high levels of sequence identities (83.6% for TaNF-YB2 and 74.9% for TaNF-YB4) to the starting bait ZmNF-YB2a.

It was previously reported that most NF-YB subunits of *Arabidopsis* can interact in Y2H assays with either the majority of or all NF-YC subunits, suggesting a low level of selectivity between NF-YB and NF-YC (Calvenzani *et al.*, 2012; Hackenberg *et al.*, 2012). In this study, however, significant selectivity in recognition of TaNF-YC15 by wheat NF-YB subunits was observed. At least three explanations can be proposed: (i) high enrichment with particular NF-Y cDNAs in the cDNA libraries (e.g. up-regulation of both isolated *NF-YB* genes by drought; Fig. 5B); (ii) high specificity of recognition between TaNF-YC15 and TaNF-YB2 or TaNF-YB4, as a result of differences in protein sequences that guide protein–protein interactions; or (iii) differences in post-translational modifications of NF-YB subunits. Identification of the same *NF-YB* genes in several different cDNA libraries and isolation of the same NF-YC subunit in reciprocal screens using TaNF-YB2 and TaNF-YB4 proteins as bait (data not shown) suggested that the specificity of NF-YB/NF-YC complex formation could be guided by a protein sequence rather than by abundance of cDNAs in libraries. It was possible to isolate a number of other *TaNF-YB* gene family members from the cDNA libraries (data not shown), but these were not identified with TaNF-YC15 in Y2H screens.

Aiming to find possible determinants of specificity in interactions between various NF-YBs and TaNF-YC15, a TaNF-YB2 segment that may be responsible for the interaction with TaNF-YC15 was mapped (Fig. 1C). A minimal size segment of TaNF-YB2, which preserves a strong ability to bind TaNF-YC15, started at residue 50 (inside the N-terminal end of the HFD) and was finished at residue 146 (downstream of the C-terminal end of the HFD). Further truncations from either side led to disruption of protein–protein interactions, because of either the deletion of key residues responsible for these interaction and/or disruption of HFD folds (Fig. 3). The minimal interacting segment of TaNF-YB2 was compared with similar segments of TaNF-YB4 and other known wheat NF-YB subunits (Fig. 3A). It was found that similarly to Gln90 of human NF-YB, Gln119 of TaNF-YB2 and the respective glutamine of TaNF-YB4 were situated close to Arg93, which was involved in TaNF-YB2/TaNF-YC15 complex formation (Fig. 3C). In all other wheat NF-YB sequences, this glutamine residue was substituted by histidine. Since histidine imidazole side chains play roles in electrostatic interactions of proteins, the histidine to glutamine substitution in TaNF-YB2 and TaNF-YB4 may directly influence the strength of interaction with TaNF-YC15 (Hansen and Kay, 2014). It is suggested that the histidine/glutamine substitution may play a critical role in the specificity of the interaction between TaNF-YC15 and TaNF-YB2 or TaNF-YB4.

### Expression of *TaNF-YB2*, *TaNF-YB4*, and *TaNF-YC15* is regulated in a similar way in most tested tissues and under drought stress, but differs under rapid dehydration

Analysis of expression levels of *TaNF-YB2*, *TaNF-YB4*, and *TaNF-YC15* revealed that all three genes are expressed in all tested tissues, indicating that their products are in close physical proximity to each other for the formation of protein complexes. However, significant differences in levels of *TaNF-YB2* and *TaNF-YB4* expression in particular tissues (e.g. mature endosperm) might suggest the possible importance of some specific features of protein sequences for the formation of particular transcriptional complexes, and hence differences in function of TaNF-YB2 and TaNF-YB4. Observations of *NF-YB2/4* gene expression in all tested wheat tissues confirm the results of wheat *NF-Y* expression analysis reported by Stephenson *et al.* (2007). Furthermore, the presence of transcripts of other NF-YB and NF-YC subunits in the same tissues (Stephenson *et al.*, 2007) and detection of these in the cDNA libraries (data not shown), together with results of the Y2H screens, advocate the fact that interactions between particular NF-YB and NF-YC subunits can be more specific than other similar interactions.

All three isolated *NF-Y* genes were up-regulated by drought. In contrast to the stress-inducible control gene *TaCor39* (Guo *et al.*, 1992; Kovalchuk *et al.*, 2013), which is progressively induced by slowly developing drought, both *TaNF-YB* and *TaNF-YC15* genes were activated at two points, initially when plants began to sense insufficiency of water, and a second time at wilting point, when water deficit became severe (Fig. 5). Maximal levels of *TaNF-YB2* activation in the present experiments were considerably lower than the level of activation of the same gene by dehydration reported by Stephenson *et al.* (2007). This may be explained by different stress conditions used in these experiments, and possibly an insufficient number of data points in the experiment for detecting the peak time of expression. In comparison, *TaNF-YB2* transcript levels were increased by 3-fold in detached leaves subjected to rapid dehydration, a similar level of induction to the stress-inducible reference gene *TaCor39* (Fig. 5). *TaNF-YB4* and *TaNF-YC15* showed very similar patterns of expression under dehydration. Both genes were initially down-regulated, then after 2h of leaf dehydration they partially returned to initial levels of expression and later again decreased by as much as 2.5-fold. These results suggest three interesting conclusions: (i) *TaNF-YB2* and *TaNF-YB4* may be induced by different components of drought stress, in the case of *TaNF-YB2* by dehydration, and therefore may play different roles in drought stress responses; (ii) significant differences in levels of expression of *TaNF-YB2* and *TaNF-YB4* in mature endosperm may be a result of *TaNF-YB2* activation by natural desiccation of grain at this stage of development; or (iii) TaNF-YB4 and TaNF-YC15 are more likely to be constituents of the same NF-Y protein complex than TaNF-YB2 and TaNF-YC15. The activation of *NF-Y* genes by drought was ABA independent, because no significant changes in expression of all three genes were induced by treatment with 200 μM ABA.

### Constitutive overexpression of *TaNF-YB4* improves performance of transgenic wheat

While constitutive overexpression of stress-related TFs generally confers improved plant reactions to corresponding stresses, in many cases it does not increase yield (seed weight per plant) (Hsieh *et al.*, 2002; Oh *et al.*, 2005, 2007; Kobayashi *et al.*, 2008*a*, *b*; Takumi *et al.*, 2008). In contrast, strong constitutive overexpression of TFs often results in development of undesirable pleiotropic phenotypes, and consequently reduces grain yields (Jaglo-Ottosen *et al.*, 1998; Liu and Zhu, 1998; Kasuga *et al.*, 1999; Hsieh *et al.*, 2002; Ito *et al.*, 2006; Morran *et al.*, 2011; Kovalchuk *et al.*, 2013). Reports of the overexpression of some NF-Y subunits in maize and rice, however, are promising exceptions to this rule (Nelson *et al.*, 2007; Wei *et al.*, 2010). The main purpose of this study was to identify and clone wheat *NF-Y* gene(s) responsible for yield increase and explore the possibility of improving performance of an elite wheat cultivar by overexpression of such gene(s).

In order to be of practical value, transgenic events need to demonstrate advantages over and above those achieved through conventional breeding. This can best be done through comparison with elite lines bred for the target environments. Evaluation of yield and yield components of transgenic plants would ideally be conducted under field conditions in large plots at multiple locations. This is costly and slow given the complex regulatory environment associated with the evaluation of transgenic plants. Some form of preliminary screening is needed to identify promising genes and transgenic events to proceed to field evaluation. However, assessing yield under controlled growth condition is difficult. In this study, transgenic lines produced in an elite wheat cultivar were generated that showed good yield under the hot, dry conditions experienced in the Mediterranean-type environment of southern Australia, where crops are grown on low nutrient soils with a strong terminal drought stress. Comparisons are commonly made between transgenic and null segregants grown in small pots, but a more challenging test is to show that the transgenics can outperform elite lines under conditions as close to the field as is practical in controlled environments. Although null segregants were included in the experiments, the comparisons are based on a non-transgenic control (WT) grown in large bins, at low nutrient status to best approximate field conditions.

Preliminary phenotyping revealed no negative influence of the constitutively expressed *TaNF-YB4* transgene on plant phenotype and yield for three independent transgenic lines showing moderate transgene expression. In contrast, line 3 showed slower growth, lower grain number, and a 10% lower grain yield compared with control plants. Levels of transgene expression in this line were ~10-fold higher than in the other lines, suggesting that limiting transgene expression to more moderate levels may be critical for avoiding deleterious phenotypes.

A replicated experiment was conducted in large containers in which soil moisture could be controlled, to simulate seasonal drought stress for one of the treatments. In this experiment, spike number, biomass, and grain yield of transgenic plants were significantly higher than for WT plants under well-watered conditions. Yield increase was observed for all five analysed T_2_ sub-lines of three transgenic lines (three independent transformation events), of up to 20–30% (Fig. 6). Yield increase in crop plants as a result of overexpression of *NF-YB* genes has been reported previously (Nelson *et al.*, 2007; Wei *et al.*, 2010). In the first report, transgenic maize plants with enhanced *ZmNF-YB2* expression demonstrated increased tolerance to drought based on improvement of parameters such as chlorophyll content, stomatal conductance, leaf temperature, reduced wilting, and maintenance of photosynthesis. These stress adaptations contributed to a grain yield advantage for transgenic maize lines under water-limited conditions (Nelson *et al.*, 2007). Although TaNF-YB4 is closely related to ZmNF-YB2, under limited water conditions, neither reduced wilting nor increased grain yield were observed in transgenic wheat plants compared with untransformed control plants. This may be explained by differences in protein sequences between ZmNF-YB2/TaNF-YB2 and TaNF-YB4, leading to formation of different complexes with other transcription factors and/or different roles for these proteins. The differences in expression patterns of *TaNF-YB2* and *TaNF-YB4* under dehydration stress also suggest diverse roles for their products under stress and during grain desiccation. It is planned to investigate the drought stress adaptation of transgenic wheat with constitutive overexpression of *TaNF-YB2*.

In another study, a rice *HAP3H* (*NF-YB*) gene, *DTH8* [quantitative trait locus (QTL) for *d*ays *t*o *h*eading on chromosome *8*], was overexpressed in rice (Wei *et al.*, 2010). It was shown that DTH8 suppresses rice flowering under long-day conditions and plays an important role in the regulation of plant height and yield potential. Although the product of the *DTH8* gene belongs to another clade of NF-YB TFs (Fig. 2A), the phenotype described for *DTH8* transgenic rice plants to some extent resembles the phenotype of *TaNF-YB4* transgenic wheat plants. Both transgenic events led to increased biomass production. *TaNF-YB4* transgenic wheat plants in the T_0_ and T_1_ generations, similarly to DTH8 rice plants, were slightly taller than control plants, although this difference in height was not so pronounced as in *DTH8* transgenic rice, and was not observed in T_2_ and subsequent generations. There are also some differences in development between *TaNF-YB4* wheat lines and *DTH8* transgenic rice lines. *DTH8* delayed flowering of transgenic rice by negatively influencing the expression of *Ehd1* and *Hd3a* genes under long-day conditions. No significant differences in flowering time of transgenic *TaNF-YB4* and control wheat plants, or changes in the dependence of flowering time on day-length, were observed (data not shown). The *TaNF-YB4* gene in transgenic wheat appears to influence tiller number rather than plant height. The increased number of tillers is responsible for higher biomass and a higher number of grains per plant. Both transgenic events produce more grain per plant with little or no loss in grain weight, and therefore numbers of grain per plant was a crucial yield component responsible for yield improvement in both cases.

Tillering in monocots is closely associated with yield. However, knowledge of the physiology and genetics of tillering is incomplete. Excessive tillering in cereal crops can lead to yield reductions because tillers compete for resources and many secondary tillers are not fertile (Kebrom *et al.*, 2012). In spite of an increased number of tillers in transgenic *TaNF-YB4* lines, most of these were fertile and reached maturity, suggesting that overexpression of *TaNF-YB4* provided an enhancement of nutrient uptake and/or more efficiency of nutrient utilization, for which the underlying mechanism is as yet unknown.

Under water-limited conditions *TaNF-YB4*-overexpressing wheat lines maintained parity in yield performance, but did not increase yield under drought conditions (Fig. 6). One of the reasons for this may be due to higher demands of wheat plants for water under drought, leading to a decline of the number of tillers. Because of the initiation of extra tillers in transgenic lines that might not occur under drought conditions, the biomass and yield of transgenic plants may remain the same as those of a control drought-tolerant cultivar with good yield-under-drought characteristics. What is important that the drought conditions applied in this experiment did not reduce grain yield of transgenic lines to levels below those of the WT, yet a significant increase of yield is observed under sufficient watering. Further experiments in both controlled conditions and multi-environment field trials are planned to investigate more thoroughly the effects of drought on *TaNF-YB4*-overexpressing wheat.

The yield improvement observed for *TaNF-YB4* transgenic lines under sufficient watering may have been a consequence of an improved ability of the transgenic plants to grow better than control plants on nutritionally poor soil. To investigate this hypothesis, an experiment was conducted where transgenic and control plants were grown in soil with different concentrations of a complete, slow-release fertilizer. The elevated concentrations of fertilizer significantly increased the yield of WT plants, although yields still remained lower than those of the transgenic lines. In contrast, the yield of transgenic plants was not boosted by fertilizer, and in fact the highest concentration of fertilizer used in the experiment negatively influenced single-grain weight (Fig. 7).

It was reported recently (Qu *et al.*, 2015) that the phenotype of transgenic wheat with constitutive overexpression of the gene encoding the NF-Y subunit A of the trimeric TF, TaNFYA-B1 was similar to that described in this work: transgenic plants produced higher grain yield than control plants because of an increased number of tillers and consequently an increased number of seeds. It was demonstrated that the increased nitrogen and phosphorus uptake may be a result of changed root morphology and up-regulated levels of nitrate and phosphate transporters in roots (Qu *et al.*, 2015). Although the present preliminary results revealed no changes in root size and morphology, it cannot be excluded that the reported TaNFYBA-B1 and currently described TaNF-YB4 interacting partners are parts of the same or a similar trimeric protein complex. It is also suggested that the constitutive up-regulation of each of the subunits may lead to the increased functional efficiency of the whole trimeric NF-Y complex. This hypothesis can explain that similar phenotypes of transgenic wheat plants were observed both in the study with *TaNFYBA-B1* (Qu *et al.*, 2015) and in the current study with *TaNF-YB4*. Further research with the transgenic lines is planned to investigate the possible mechanisms by which *TaNF-YB4*-overexpressing wheat plants overcome nutrient deficiency in soil.

## Supplementary data

Supplementary data are available at *JXB* online.

Figure S1. Confirmation of transgene (*TaNF-YB4*) expression in transgenic wheat lines.

Figure S2. Large container systems used for plant growth (soil-water potential and outlook of containers).

Table S1. List of PCR primers used in this study.

Supplementary data. List of GenBank accession numbers of protein sequences used for construction of phylogenetic trees (Fig. 2).

Supplementary Data
