# Supplementary material for: Constitutive overexpression of the TaNF-YB4 gene in transgenic wheat significantly improves grain yield
Source: J Exp Bot. 2015 Jul 27;66(21):6635–50. doi: 10.1093/jxb/erv370 (PMC4623681; doi:10.1093/jxb/erv370)
Supplement: Supplementary Data [file supp_66_21_6635__index.html]

Constitutive overexpression of the TaNF-YB4 gene in transgenic wheat significantly improves grain yield — Constitutive overexpression of the TaNF-YB4 gene in transgenic wheat significantly improves grain yield — Constitutive overexpression of the TaNF-YB4 gene in transgenic wheat significantly improves grain yield — Supplementary Data 

# Constitutive overexpression of the *TaNF-YB4* gene in transgenic wheat significantly improves grain yield

## Supplementary Data

Data files

- Supplementary Data - Supplementary Data
