# Supplementary material for: Constitutive overexpression of the TaNF-YB4 gene in transgenic wheat significantly improves grain yield
Source: J Exp Bot. 2015 Jul 27;66(21):6635–50. doi: 10.1093/jxb/erv370 (PMC4623681; doi:10.1093/jxb/erv370)
Supplement: Supplementary Data [file supp_erv370_jexbot149088_file001.pdf]

Constitutive over-expression of the *TaNF-YB4* gene in transgenic wheat significantly improves grain yield

Dinesh Yadav, Yuri Shavrukov, Natalia Bazanova, Larissa Chirkova, Nikolai Borisjuk, Nataliya Kovalchuk, Ainur Ismagul, Boris Parent, Peter Langridge, Maria Hrmova, Sergiy Lopato

Supplementary material

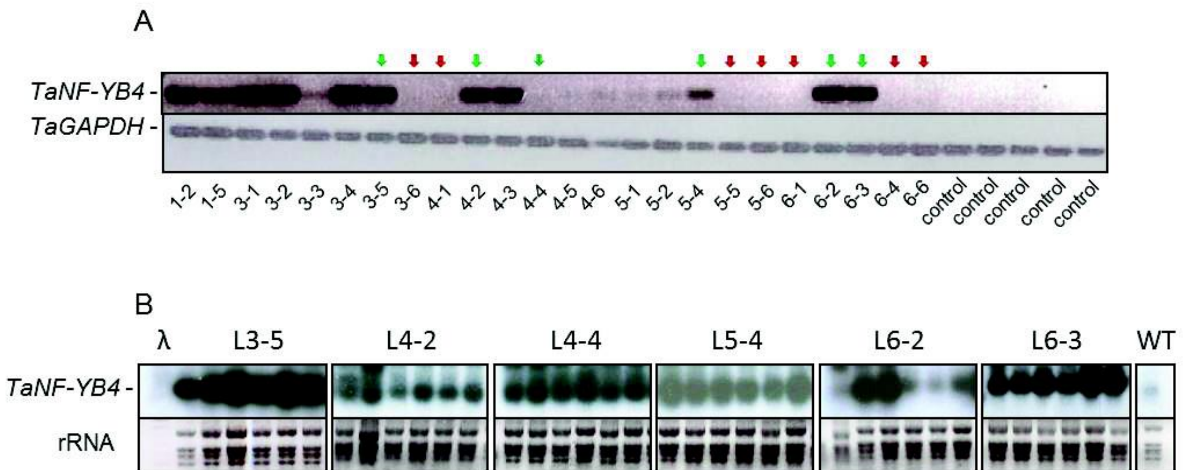

Figure S1. Confirmation of transgene (*TaNF-YB4*) expression in transgenic wheat lines. **(A)** Transgene expression monitored by RT-PCR in T_1 transgenic plants. The *Glyceraldehyde 3 Phosphate Dehydrogenase* (*GAPDH*) gene was used as a control reference gene. Red arrows indicate null-segregants confirmed for each plant by q-PCR-based data on copy numbers. Green arrows indicate T_1 plants selected for the analysis of T_2 progeny. RT-PCR data were confirmed by Northern blot hybridization (results not shown here). **(B)** Transgene expression monitored by Northern blot hybridization in T_2 transgenic lines. Six plants from each T_1 progeny were randomly selected for this panel. Wild type (WT) plant was used as negative control.

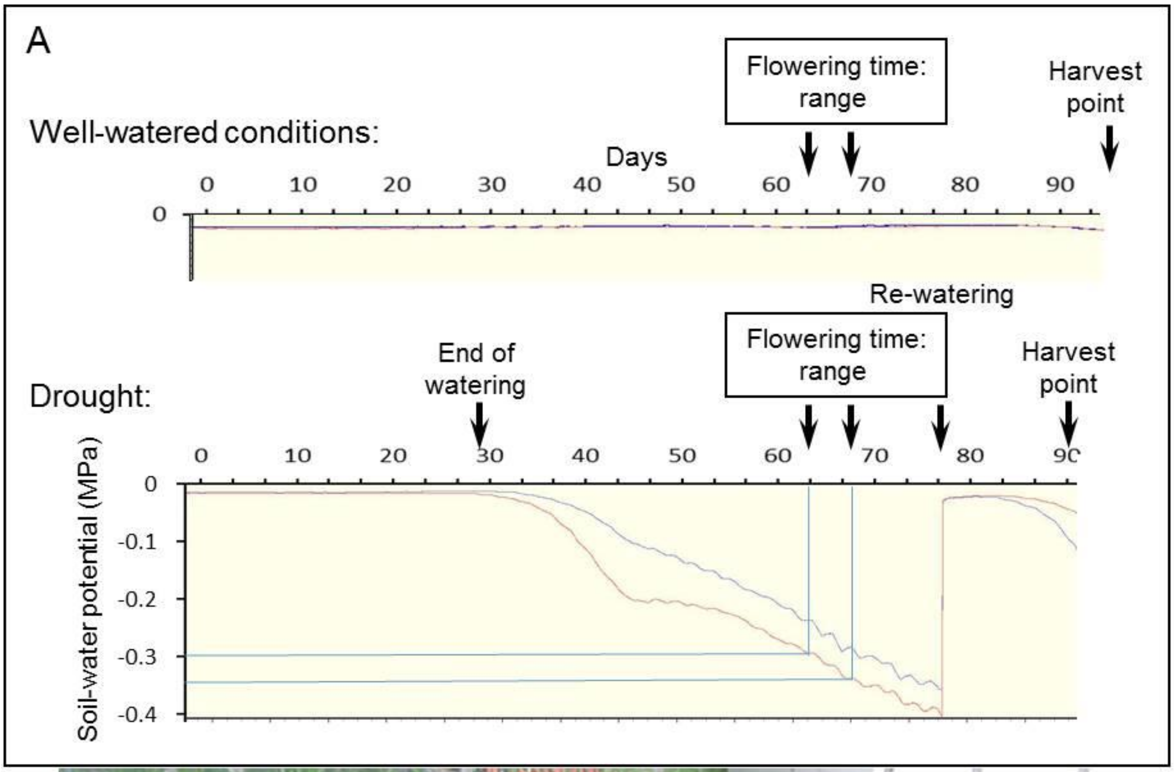

B
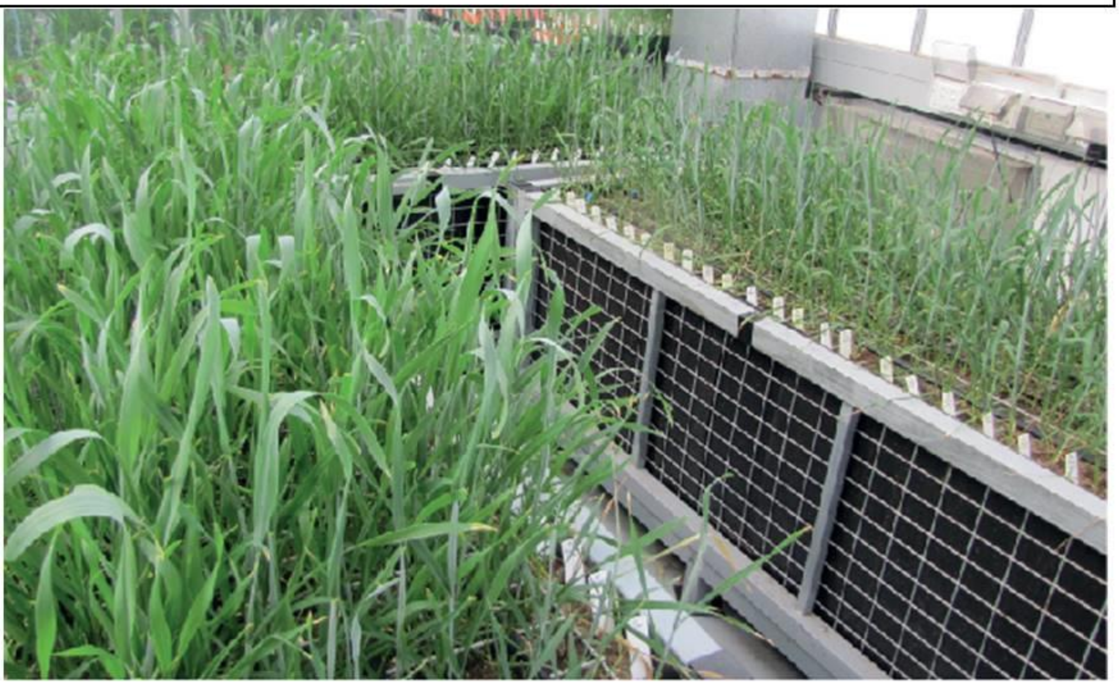

Figure S2. Large container systems used for plant growth. **(A)** The soil-water potential shown by two sensors situated near the bottom of the container and just under the soil surface, in well-watered growth conditions and under drought. **(B)** Outlook of containers.

Supplementary data

NF-YB entries used for Figure 2

TaNF-YB1 (BT009029, TaNF-YB2 (BT009078), TaNF-YB3 (BT009265), TaNF-YB4 (BT009393), TaNF-YB5 (CK203103), TaNF-YB6 (CV776390), TaNF-YB7 (CK213790), TaNF-YB8 (CJ724868), TaNF-YB11 (CJ856713), TaNF-YB12 (GH723061), OsHAP3A (BAC76331), OsHAP3B/OsNF-Y3B (BAC76332), OsHAP3C (BAC76333), OsHAP3E (BAF6443), OsHAP3F (BAF64445), OsHAP3J (FAA00426), OsHAP3I (BAF64448), OsHAP3H/DTH8 (BAF64447), OsHAP3G (BAF64446), OsHAP3K/LEC1-like (AAL47204), OsNF-YB1 (CAC37695), AtNF-YB1/HAP3A (NP_030436), AtNF-YB2 (NP_199575), AtNF-YB3/HAP3C (AEE83458), AtNF-YB4 (AEE28385), AtNF-YB5 (AEC10890), AtNF-YB6/LIL (AED95548), AtNF-YB7 (AEC06243), AtNF-YB8 (AEC06243), AtNF-YB9/LEC1 (AEE30179), AtNF-YB10 (AEE79070), AtNF-YB13 (CBW53706), ZmNF-YB1 (CBW53688), ZmNF-YB2 (NP_001106052), ZmNF-YB2a (NP_001105435), ZmNF-YB3 (NP_001147638), ZmNF-YB4 (NP_001152278), ZmNF-YB5 (NP_001266909), ZmNF-YB6 (DAA53600), ZmNF-YB7 (CBW53701), ZmNF-YB8 (AFW63719), ZmNF-YB9 (CBW53696), ZmNF-YB10 (NP_001152628), ZmNF-YB11 (CBW53694), ZmNF-YB12 (CBW53703), ZmNF-YB13 (DAA53600), ZmNF-YB14 (DAA59933), ZmNF-YB15 (CBW53702).

NF-YC entries used for Figure 2

TaNF-YC1 (GH7314), TaNF-YC2 (BT008988), TaNF-YC3 (BT009224), TaNF-YC4 (DN829033), TaNF-YC5 (DR738968), TaNF-YC6 (CJ855361), TaNF-YC7 (CV762585), TaNF-YC8 (CD883696), TaNF-YC10 (BT008988), TaNF-YC11 (CD888515), TaNF-YC13 (BJ308764), OsHAP5A (BAF64449), OsHAP5B (BAF64450), OsHAP5C (BAF64451), OsHAP5D (BAF64452), OsHAP5E (BAF64453), OsHAP5F (BAF64454), OsHAP5G (BAF64455), AtNF-YC1 (AEE78434), AtNF-YB2 (AEE33354), AtNF-YC3 (AEE33153), AtNF-YC4 (AED97756), AtNF-YC5 (AED95951), AtNF-YC6 (AED959550), AtNF-YC7 (AED95949), AtNF-YC8 (AED93743), AtNF-YC9 (AEE28377), AtNF-YC10 (AEE28222), AtNF-YC11 (AEE75200), AtNF-YC12 (AED94272), AtNF-YC13 (AED94931).

Table S1. List of PCR primers used in this study.

Short name	Purpose	Forward primer	Reverse primer
ZmNF-YB2	Cloning in the Y2H vectors	GAAGAATTCATGGCGGAAGCTCCGGCGAG	GGAGGATTCCCATTAGTTTGAGATATCC
TaNF-YB2	Cloning in the Y2H vectors	GAAGAATTCATGTCGGACGAGGCGGCGAG	GGAGGATCCTCAGTTTGAGATGTCCCCATTATGGTACT
TaNF-YB4	Cloning in the Y2H vectors	GAAGAATTCATGGCCGACGACGACAG	GGAGGATCC TCAGGTGTCCCCATTATGGTAC
TaNF-YC15(d1)	Cloning in the Y2H vectors	GAAGAATTCTTCTGGGCCGAACGACTG	GAAGGATCCTCAGCCGCTTCCAGATTG
TaNF-YB4	Cloning in the pENTR-D-TOPO	CACCATGGCCGACGACGACAG	TCAGGTGTCCCCATTATGGTAC
TaNF-YC15	Q-PCR, gene expression	GATTCAGCATCGTCAGCTTAG	TTGTAAGGGCACCACCACCAC
TaNF-YB2	Q-PCR, gene expression	TCCTAGGTGGGTCATCATGTG	AGACAACAACAACAACCATGC
TaNF-YB4	Q-PCR, gene expression	GGACACCTGAAACTGAAGATC	GCCATCACAACCAACTGTTTC
TaActin	Q-PCR, normalisation	GACAATGGAACCGGAATGGTC	GTGTGATGCCAGATTTTCTCCAT
TaCyclophilin	Q-PCR, normalisation	CAAGCCGCTGCACTACAAGG	AGGGGACGGTGCAGATGAA
TaGAPdH	Q-PCR, normalisation	TTCAACATCATTCCAAGCAGCA	CGTAACCCAAAATGCCCTTG
TaEFa	Q-PCR, normalisation	CAGATTGGCAACGGCTACG	CGGACAGCAAAACGACCAAG
TaNF-YB4	Transgene specific primers	GGCCAACGGCAAGATCGCCAAG	TCTAGTAACATAGATGACACC
Nos terminator	Q-PCR, copy number	CTTAAGATTGAATCCTGTTGCCGGTC	CGAATTCAGTAACATAGATGACACCGC
TaNF-YB2	Y2H, CDS	GAAGAATTCATGGCGGAAGCTCCGGCGAG	GGAGGATTCCCATTAGTTTGAGATATCC
TaNF-YB2D1	Y2H, deletions	GAAGAATTCGGCGTCAGGGAGCAGGACAG	GGAGGATTCCCATTAGTTTGAGATATCC
TaNF-YB2D2	Y2H, deletions	GAAGAATTCATGAAGAAGGCCATCC	GGAGGATTCCCATTAGTTTGAGATATCC
TaNF-YB2D3	Y2H, deletions	GAAGAATTCCAGGAGTGCGTCTCCGAGT	GGAGGATTCCCATTAGTTTGAGATATCC
TaNF-YB2D4	Y2H, deletions	GAAGAATTCGACAAGTGCCAGGG	GGAGGATTCCCATTAGTTTGAGATATCC
TaNF-YB2D5	Y2H, deletions	GAAGAATTCGACGACCTGCTCTG	GGAGGATTCCCATTAGTTTGAGATATCC
TaNF-YB2D6	Y2H, deletions	GAAGAATTCCAGAAGTACAGAGAGA	GGAGGATTCCCATTAGTTTGAGATATCC
TaNF-YB2D7	Y2H, deletions	GAAGAATTCGATGCACTTGGTCCTC	GGAGGATTCCCATTAGTTTGAGATATCC
TaNF-YB2D8	Y2H, deletions	GAAGAATTCATGGCGGAAGCTCCGGCGAG	GGAGGATTCTACTTGTTGGCCCATC
TaNF-YB2D9	Y2H, deletions	GAAGAATTCATGGCGGAAGCTCCGGCGAG	GGAGGATTCGAGGACCAAGTGCATC
TaNF-YB2D10	Y2H, deletions	GAAGAATTCATGGCGGAAGCTCCGGCGAG	GGAGGATTCCTCTCTGTACTTCTG
TaNF-YB2D11	Y2H, deletions	GAAGAATTCATGGCGGAAGCTCCGGCGAG	GGAGGATTCGATGTACTCCTCGAAG
TaNF-YB2D12	Y2H, deletions	GAAGAATTCATGGCGGAAGCTCCGGCGAG	GGAGGATTCCCAGAGCAGGTCGTC
TaNF-YB2D13	Y2H, deletions	GAAGAATTCATGGCGGAAGCTCCGGCGAG	GGAGGATTCGGAGACGCACTCCTGCAC
TaNF-YB2D14	Y2H, deletions	GAAGAATTCATGGCGGAAGCTCCGGCGAG	GGAGGATTCGTCCTTGGCGATCTTGC
TaNF-YB2D15	Y2H, deletions	GAAGAATTCATGGCGGAAGCTCCGGCGAG	GGAGGATTCGATGGCCTTCTTCATG
